# Analytical Separation of Carcinogenic and Genotoxic Alkenylbenzenes in Foods and Related Products (2010–2020)

**DOI:** 10.3390/toxins13060387

**Published:** 2021-05-28

**Authors:** Huynh N. P. Dang, Joselito P. Quirino

**Affiliations:** Australian Centre for Research on Separation Science (ACROSS), School of Natural Sciences—Chemistry, University of Tasmania, Hobart, TAS 7001, Australia; huynhngocphuong.dang@utas.edu.au

**Keywords:** alkenylbenzenes, food, liquid chromatography, gas chromatography, capillary electrophoresis, sample preparation

## Abstract

Alkenylbenzenes are potentially toxic (genotoxic and carcinogenic) compounds present in plants such as basil, tarragon, anise star and lemongrass. These plants are found in various edible consumer products, e.g., popularly used to flavour food. Thus, there are concerns about the possible health consequences upon increased exposure to alkenylbenzenes especially due to food intake. It is therefore important to constantly monitor the amounts of alkenylbenzenes in our food chain. A major challenge in the determination of alkenylbenzenes in foods is the complexity of the sample matrices and the typically low amounts of alkenylbenzenes present. This review will therefore discuss the background and importance of analytical separation methods from papers reported from 2010 to 2020 for the determination of alkenylbenzenes in foods and related products. The separation techniques commonly used were gas and liquid chromatography (LC). The sample preparation techniques used in conjunction with the separation techniques were various variants of extraction (solvent extraction, liquid-liquid extraction, liquid-phase microextraction, solid phase extraction) and distillation (steam and hydro-). Detection was by flame ionisation and mass spectrometry (MS) in gas chromatography (GC) while in liquid chromatography was mainly by spectrophotometry.

## 1. Introduction

Throughout human history, plants and their constituents have been used as a source of medicine [1]. The availability and health benefits of plants also allow them to become part of the common human diet; thus, they are found as ingredients in many foods and food/health supplements [2]. However, not all chemicals in plants (phytochemicals) are associated with positive health effects and many of these natural chemicals are potentially toxic. Rietjens, et al. [3] have listed out several phytochemicals that are (or potentially) toxic such as aristolochic acids, pyrrolizidine alkaloids, coumarin, ephedrine alkaloids, synephrine, kavalactones, β-carotene, anisatin, solanine, chaconine, thujone, cyanogenic glycosides and glycyrrhizinic acid. The focus of this review is on the alkenylbenzenes (especially the carcinogenic and genotoxic alkenylbenzenes) that are naturally found in many plants and used in food and food/health related products. A brief introduction and importance of the different aspects of analytical methods for their determination will be highlighted.

Alkenylbenzenes are present in many edible consumer products including spices, plant food supplements and herbal medicines. The most common spices containing alkenylbenzenes are anise star, basil, black pepper, cinnamon, coriander, dill, nutmeg, parsley and tarragon. In particular, estragole is present in significant amounts in fennel [4] while methyleugenol in basil and nutmeg [5]. These spices are used in alcoholic and non-alcoholic beverages, and in preparing baked goods and sweets [6]. Safrole in particular is known for its ‘candy shop’ aroma [7,8].

**Figure 1:**
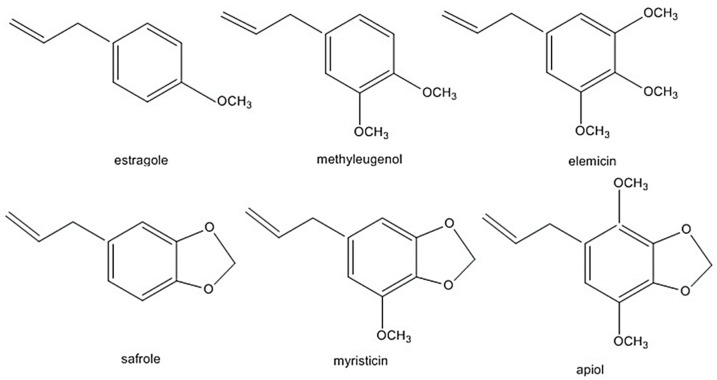
Alkenylbenzenes that are genotoxic and carcinogenic. Reprinted with permission from the Authors [13].

The alkenylbenzenes apiol, elemicin, estragole, methyleugenol, myristicin, and safrole have been considered toxic [9,10,11,12] (structures are shown in Figure 1). Safrole had been prohibited in the US since 1960 while estragole and methyleugenol are currently being revaluated as safe (generally recognised as safe status). In addition, in the light of exposure estimates from all sources, the European Union (EU) Scientific Committee on Food (EU-SCF) concluded that estragole, methyleugenol and safrole fall in the priority for risk management and are considered genotoxic and carcinogenic. Therefore, restrictions on using these compounds as food additive is in place [9,10,11]. Nevertheless, there have been reports that some foods and beverages contain levels higher than the maximum levels of safrole. For example, cola beverages may contain up to 4.5 mg/kg of safrole, which is 4× the EU maximum level [7,8]. The EU maximum level is 1 mg/kg of safrole in foods and beverages. In the case of methyleugenol and estragole, the EU-SCF cannot establish their exposure limits on food [9,11]. Clearly, more studies are required to clarify exposure limits which will influence legal regulations of these phytochemicals.

All phytochemicals are possibly toxic and large doses can lead to poisoning effects [3]. Chemical risk assessment aims to quantify chemical exposures from food for a given human population. It then determines safe chemical levels and quantifies the risk associated with the exposures. An essential aspect of chemical risk assessment is to obtain dependable occurrence data on the phytochemicals in food. Occurrence data relies on the availability and reliability of analytical methods that can handle complex food matrices and quantify the phytochemicals at mostly low concentration levels. The objective of this review is to discuss the background and applications of the analytical methods (sample preparation and separation) used for the determination of alkenylbenzenes, especially in foods and related products.

To conduct a comprehensive research on alkenylbenzenes studies, Scopus database was used. The search parameter involved different sets of keywords “alkenylbenzenes”, “methyleugenol”, “myristicin”, “estragole”, “safrole” and “analytical separation technique”, and the year of publication was limited between 2010 to 2020. Liquid chromatography (LC), gas chromatography (GC) or capillary electrophoresis (CE) are the analytical separation techniques. There were >20 research papers, and most papers were on GC and LC. The latest study conducted using CE was in 2008, which was also included in this review. Sample preparation is an important step in the analysis, thus the extraction and clean-up methods reported in the considered papers were categorised and discussed. For GC, these were liquid- liquid extraction (LLE), liquid-phase microextraction (LPME), Quick Easy Cheap Effective Rugged Safe (QuEChERS), distillation (steam and hydro-), and solid phase extraction (SPE). Interestingly, only solvent extraction (primarily with methanol) was reported in combination with HPLC.

## 2. Gas Chromatography

### 2.1. Analysis

Gas chromatography was invented in 1952 by A. T. James and A. J. P. Martin. GC is a simple and fast analytical separation technique that is applicable to many volatile compounds (with high vapour pressure at temperature below 350 °C to 400 °C) such as alkenylbenzenes [14]. It utilises solid or liquid as stationary phase and gas as mobile phase. A typical gas chromatography system consists of carrier gas (mobile phase), injector, GC column (stationary phase), detector and data system. GC operates by injecting a sample into one end (inlet) of the column. The mobile phase is passed through the inlet and carries the sample onto the column. The separation occurs while the sample travels through the column, which is temperature controlled. When the sample exits the column, it enters the detector where it produces an electronic signal [15]. The results are plotted in a so-called chromatogram.

Table 1 summarizes the papers that used GC for the determination of alkenylbenzenes in different foods and related samples. The amount of alkenylbenzenes found in sample/s, sample preparation method, GC conditions, detector used, and references were included in the table. Flame ionisation detector (FID) and mass spectrometry (MS) are the dominant detectors for quantitation. FID has several advantages such as high sensitivity, low noise level and a wide linear range. However, FID cannot unambiguously identify the peaks in GC chromatograms or distinguish components in overlapping peaks [16]. On the other hand, MS is a very powerful detector as it can be used to identify the alkenylbenzenes at a molecular level.

For high-sensitivity analysis with confident identification of alkenylbenzenes, a few studies combined FID with MS [17,18,19]. Meanwhile, Rivera-Pérez, et al. [20] developed a GC method with high-resolution mass spectrometry (HRMS) and Q-Orbitrap. They believed HRMS was highly accurate and allowed retrospective analysis. It can elucidate unknown components in complex matrices due to its highly sensitive full-scan mode. In addition, Q-Orbitrap was able to separate isobaric compounds. They concluded that GC with HRMS and Q-Orbitrap enabled post-targeted analysis of other alkenylbenzenes compounds without having to reanalyse them.

**Figure 2:**
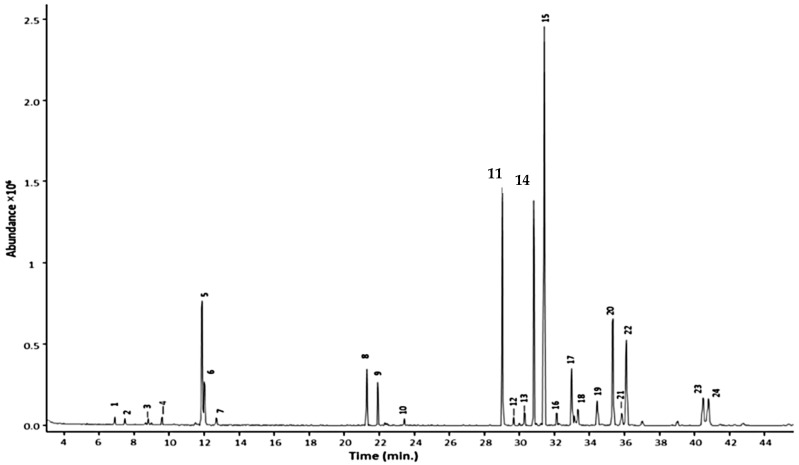
GC chromatogram of Ocimum micranthum Willd leaves’ essential oil obtained with a capillary column coated with a mid-polar stationary phase 50%-phenyl-methylpolysiloxane. Peaks: eugenol (11); methyleugenol (14). Reprinted with permission from the Authors [21].

In terms of GC conditions (see Table 1), majority of studies used helium as the carrier gas and only one study used nitrogen. Vaporisation injection with temperatures between 230 °C to 300 °C were implemented. Open-tubular capillary columns that provided high separation performance were used rather than packed columns. The open-tubular capillary column was fused silica with inner diameter of 0.25–0.32 mm. The inner surface of a capillary was coated with a stationary phase with film thickness of 0.1–5 µm. The stationary phases varied in polarity, non-polar to highly polar. A nice example is the work of Caamal-Herrera, et al. [21], where they used a ZB-5HT INFERNO column coated with 5% phenyl 95% polydimethylsiloxane. The GC chromatogram of the essential oil of Ocimum micranthum Willd leaves using this mid-polar coated stationary phase is shown in Figure 2. The targeted alkenylbenzenes eugenol and methyleugenol was successfully separated from the other components in the multi-component essential oil with high separation efficiencies and sharp peaks.

**Table 1:** Application of GC for the determination of alkenylbenzenes.

Alkenylbenzenes	Sample/s	Sample Preparation	Amount of Alkenylbenzenes Found in Sample/s	GC Conditions	Detector	Ref.
myristicin elemicine cis-isosafrole borneol caryophyllene	*Chinese Ainsliacea fragrans* Champ ex Benth	hydrodistillation	myristicin (41.3%) elemicine (11.9%) cis-isosafrole (11.5%) borneol (9.1%) caryophyllene (8.8%) - % values were the % found in the extracted essential oil - the essential oil was 0.06% *v/w* of sample	column:HP-5, 5% diphenyl and 95% dimethylpolysiloxane, 30 m × 0.25 mm × 0.25 µm program:initial temperature at 60 °C hold for 1 min, ramp to 180 °C (10 °C/min), hold for 1 min, ramp to 280 °C (20 °C/min), hold for 15 min. carrier gas:helium, 1 mL/min injection:split, 1:10, 1 µL, 270 °C run time: 34.0 min LOD: not reported LOQ: not reported	FID	[22]
safrole apiol myristicin	essential oils from Vietnam	hydrodistillation	safrole (38.1%) apiol (10.8%) myristicin (8.0%) - % values were the % found in the extracted essential oil - the essential oil was 0.2% *v/w* of sample	column: SE-52 capillary column, 50 m × 0.25 mm × 1.0 µm program: 60 °C for 1 min, heating to 230 °C (3 °C/min), hold for 12.3 min carrier gas: helium, 1.5 mL/min injection:split/splitless, 1:100, 0.1 µL, 230 °C run time: 70.0 min LOD:not reported LOQ:not reported	FID	[23]
eugenol methyleugenol	holy basil essential oils	hydrodistillation	eugenol (37–45%) methyleugenol (65%) - % values were the % found in the extracted essential oil - the essential oil was 7.9 ± 3.2 mg/g of sample	column:HP-5 fused silica capillary, 5% phenyl methylpolysiloxane, 30 m × 0.32 mm, 0.25 µm program:initial temperature 50 °C held for 5 min, increase to 120 °C (3 °C/min), to 250 °C (5 °C/min), to 300 °C (15 °C/min) and hold for 5 min. carrier gas:helium, 25 mL/min injection: split, 50:1, 250 °C run time: 62.6 min LOD: 0.21 µg/mL LOQ: 0.54 µg/mL	FID	[18]
			MS for identification only	column:HP-5 MS fused-silica capillary column, 5% phenyl methylpolysiloxane, 30 m × 0.25 mm i.d × 0.25 µm program:50 °C for 5 min, increase to 120 °C (3 °C/min), to 250 °C (5 °C/min), hold for 0.67 min. carrier gas:helium, 25 mL/min injection:split, 50:1, 1 µL, 250 °C run time:55.0 min LOD:not reported LOQ:not reported	MS	
eugenol estragole	*Ocimum* species	hydrodistillation	eugenol (566.8 ± 98.0 mg/g–859.3 ± 151.3 mg/g) estragole (448.8 ± 126.8 mg/g–640.2 ± 44.8 mg/g)	column: RTX-5 column, methylpolysiloxane, 30 m × 0.25 mm × 0.25 µm program:initial temperature at 70 °C, heating ramp up to 180 °C (4 °C/min), ramp at 250 °C (10 °C/min) carrier gas:nitrogen, 1 mL/min injection:split, 1:30, 1 µL, 250 °C run time:34.5 min LOD:1.2 µg/mL LOQ:not reported	FID	[17]
			MS was used for identification only	column:HP-5MS column, methylpolysiloxane, 30 m × 0.25 mm × 0.25 µm program:initial temperature at 70 °C, heating ramp up to 180 °C (4 °C/min), ramp at 250 °C (10 °C/min) carrier gas: helium, 1 mL/min injection: split, 1:100, 1 µL, 250 °C run time: 34.5 min LOD: not reported LOQ: not reported	MS	
16 alkenylbenzenes	essential oils	not described	different % values of alkenylbenzenes in 23 essential oils	column: capillary column 30 m × 0.32 mm × 0.25 µm program:hold at 40 °C for 2 min, increase from 40 °C to 200 °C (10 °C/min), to 300 °C (20 °C/min). carrier gas: nitrogen, 2.1 mL/min injection:split, 1:50, 300 °C run time: 31.0 min LOD:not reported LOQ:not reported	FID	[19]
	human serum	not described	eugenol (222 ± 34 ng/mL), geraniol (6.18 ± 0.67 ng/mL), methyleugenol (0.74 ± 0.08 ng/mL), cis-isoeugenol (1.87 ± 0.69 ng/mL), acetyl eugenol (30.2 ± 11 ng/mL), myristicin (12.8 ± 1.6 ng/mL) after administration of clove essential oil cream			
	essential oils and human serum	not described	MS was used for identification only	column:DB-5 fused silica capillary column, 5% phenyl methylpolysiloxane, 30 m × 0.25 mm i.d × 0.25 µm program:increase from 45 °C to 250 °C (5 °C/min) carrier gas:helium, 0.82 mL/min injection: not reported run time:41 min LOD: not reported LOQ: not reported	MS	
methyleugenol	*Cymbo-pogon khasia-nus* Hack.	hydrodistillation	methyleugenol (73.2%) - % values were the % found in the extracted essential oil - the essential oil was 0.73% *v/w* of sample	column: HP-5 fused silica capillary, 30 m × 0.25 mm × 0.25 µm program:initial temperature at 40 °C, hold for 2 min, increase to 250 °C (5 °C/min), to 300 °C (30 °C/min), hold for 10 min carrier gas: helium, 1 mL/min injection:split, 1:20, 1 µL, 250 °C run time:55.7 min LOD:not reported LOQ:not reported	MS	[24]
methyleugenol	*Melaleuca alternifolia* oils	solvent dilution	methyleugenol (160.0 µg/mL–552.0 µg/mL)	column: Varian Factor Four VF-5, 30 m × 0.25 mm × 0.25 µm program: 130 °C to 180 °C (15 °C/min), increase to 230 °C (30 °C/min), hold for 4 min carrier gas:helium, 1.2 mL/min injection:split, 7:1, 1 µL, 240 °C run time:9.0 min LOD: 150 ppb LOQ: 500 ppb	MS	[25]
anethole estragole eugenol methyleugenol safrole myristicin	aroma-therapy massage oil products	dispersive liquid-liquid microextraction (DLLME), dual DLLME	anethole (up to 862.1 µg/g) estragole (up to 0.7 µg/g) eugenol (0.5 µg/g–851.5 µg/g) methyleugenol (0.1 µg/g–0.5 µg/g) safrole (up to 0.2 µg/g) myristicin (up to 0.7 µg/g)	column:VF-5MS fused silica capillary column, 30 m × 0.25 mm × 0.25 µm program:initial temperature at 90 °C for 1 min, ramp to 130 °C (40 °C/min), ramp to 137 °C (3.5 °C/min), ramp to 139.4 °C (0.3 °C/min), ramp to 280 °C (70 °C/min), hold for the remaining time carrier gas: helium, 2 mL/min injection: split, 10:1, 1 µL, 260 °C run time: 16.0 min LOD: 1.0–3.0 ng/mL LOQ: 2.5–10.0 ng/mL	MS	[26]
eugenol methyleugenol	*Ocimum micran-thum*	hydrodistillation	eugenol (12%) methyleugenol (14%) of the total area in the chromatogram of the distillate	column: ZB-5HT INFERNO, 5% phenyl 95% polydimethylsiloxane, 30 m × 0.25 mm i.d × 0.25 µm program: 60 °C to 68 °C (0.7 °C/min), hold for 7 min, increase to 100 °C (10 °C/min), to 130 °C (5 °C/min), hold for 7 min, to 135 °C (1 °C/min) and hold for 6 min carrier gas:helium, 1 mL/min injection:split, 1:100, 1 µL, 280 °C run time: 45.6 min LOD: not reported LOQ:not reported	MS	[21]
estragole eugenol	basil species and pot cultures	steam distillation	estragole (2.3 mg/mL in Lettuce Leaf) eugenol (1.2 mg/mL in Mammolo Genovese, 0.4 mg/mL in Manes)	column: DB-WAX, 30 m × 0.25 mm × 0.25 µm program: 40 °C for 3 min, increase to 60 °C (8 °C/min), to 70 °C (5 °C/min), to 230 °C (4 °C/min), keep for constant for 1 min. carrier gas: helium, 0.5 mL/min injection:split, 1:100, 1 µL, 240 °C run time:48.5 min LOD:0.0085 mg/mL (estragole), 0.0063 mg/mL (eugenol) LOQ:0.0118 mg/mL (estragole), 0.0066 mg/mL (eugenol)	MS	[27]
estragole methyleugenol safrole	food and beverage samples	QuEChERS	estragole (0.7 mg/kg–5.2 mg/kg in fish samples) methyleugenol (0.6 mg/kg–3.3 mg/kg in bakery, meat, dairy and vegetable samples) safrole (up to 2.4 mg/kg in butter with spices sample)	column: REStek Rtx^®^- CLPesticides, 30 m × 0.25 mm × 0.25 µm program: 60 °C for 1 min, increase to 80 °C (50 °C/min), to 125 °C (3 °C/min), to 300 °C (10 °C/min), hold for 5 min carrier gas: helium, 1 mL/min injection: splitless, 1 µL, 250 °C run time: 38.9 min LOD:not reported LOQ: 0.05 mg/kg (non-alcoholic beverages), 0.5 mg/kg (other food matrices)	MS	[28]
methyleugenol estragole	*Anthriscus cerefolium* L. Hoffm	hydrodistillation	methyleugenol (47.2% in El-Sharkia essential oil) El-Sharkia essential oil is 0.075–0.083 mL/plant estragole (18.0% in El-Fayoum essential oil) El-Fayoum essential oil is 0.12–0.16 mL/plant	column: TG-WAX, 30 m × 0.25 mm × 0.25 µm program: 40 °C for 1 min, increase to 160 °C (4 °C/min), hold for 6 min, increase to 210 °C (6 °C/min), hold for 1 min. carrier gas: helium, 1 mL/min injection: split, 1:10, 0.2 µL, 210 °C run time: 46.3 min LOD: not reported LOQ: not reported	MS	[29]
eugenol isoeugenol methyleugenol	fish fillet	solvent extraction and solid phase extraction (SPE)	eugenol (259.0 µg/kg–2329.0 µg/kg) isoeugenol (86.2 µg/kg–1032.0 µg/kg) methyleugenol (not found)	column:DB-17 capillary column, 30 m × 0.25 mm × 0.25 µm program:initial temperature at 80 °C, hold for 2 min, increase to 220 °C (25 °C/min) and hold for 1 min, increase to 280 °C (30 °C/min), hold for 1 min carrier gas: helium, 2 mL/min injection:split, 1 µL, 260 °C run time:11.6 min LOD: 0.4 µg/kg (eugenol), 1.2 µg/kg (isoeugenol), 0.2 µg/kg (methyleugenol) LOQ: 1.2 µg/kg (eugenol), 4 µg/kg (isoeugenol), 0.7 µg/kg (methyleugenol)	MS	[30]
methyleugenol	food samples	QuEChERS	methyleugenol (6.1 ± 0.4 mg/kg)	column: DB-1 capillary column, 30 m × 0.25 mm × 0.25 µm program: 70 °C for 1 min, increase to 120 °C (40 °C/min), to 180 °C (8 °C/min), hold for 1 min, to 280 °C (40 °C/min) and hold for 1 min carrier gas: not reported injection:splitless, 2 µL, 280 °C run time: 14.3 min LOD:20 µg/kg (solid/semi-solid food samples), 0.4 µg/kg (beverages) LOQ: 50 µg/kg (solid/semi-solid food samples), 1 µg/kg (beverages)	MS	[31]
methyleugenol estragole	food samples	liquid-liquid extraction	methyleugenol (4288.0 mg/kg for allspice pimento, 1351.0 mg/kg for nutmeg and n.d–1184.0 mg/kg for basil) estragole (not reported)	column: HP-Innowax, fused silica capillary column, 41 m × 0.25 mm × 0.25 µm program: 40 °C for 1 min, increase to 200 °C (8 °C/min), hold for 5 min carrier gas: helium, 1 mL/min injection:split, 1:5, 1 µL, 240 °C run time: 26.0 min LOD: 2.1 mg/L (methyleugenol), 1.3 mg/L (estragole) LOQ: 5.3 mg/L (methyleugenol), 4.7 mg/L (estragole)	MS	[32]
estragole tr-anethole safrole eugenol tr-iso-eugenol acetyl eugenol methyleugenol myristicin	pepper and its varieties	ultrasound-assisted extraction	estragole (2.2 mg/kg–45.7 mg/kg) tr-anethole (10.7 mg/kg–42.7 mg/kg) safrole (0.2 mg/kg–3.0 mg/kg) eugenol (10.5 mg/kg–120.0 mg/kg) tr-iso-eugenol (0.7 mg/kg–3.6 mg/kg) acetyl eugenol (45.8 mg/kg in red pepper) methyleugenol (0.5 mg/kg–20.1 mg/kg) myristicin (0.2 mg/kg–6.1 mg/kg)	column: BP5MS capillary analytical, 30 m × 0.25 mm × 0.25 µm program:initial temperature at 70 °C (3 min), increase from 70 °C to 250 °C (10 °C/min) then increase to 280 °C (50 °C/min) and maintain for 3 min carrier gas: helium, 1 mL/min injection:splitless, 2 µL, 280 °C run time: 24.6 min LOD:0.02 mg/kg (estragole), 0.02 mg/kg (trans-anethole), 0.01 mg/kg (safrole), 0.01 mg/kg (eugenol), 0.01 (trans-iso-eugenol), 0.01 mg/kg (acetyl eugenol), 0.01 mg/kg (methyleugenol), 0.01 mg/kg (myristicin) LOQ:0.2 mg/kg (estragole), 0.2 mg/kg (trans-anethole), 0.2 mg/kg (safrole), 0.2 mg/kg (eugenol), 0.2 (trans-iso-eugenol), 0.2 mg/kg (acetyl eugenol), 0.2 mg/kg (methyleugenol), 0.2 mg/kg (myristicin)	HRMS-Q-Orbitrap	[20]

### 2.2. Sample Preparation

In GC, the sample is vaporised and injected into a port at the head of GC column. Food samples are not amenable to directly injection into GC columns; therefore, sample preparation is essential prior to analysis. The aim of sample preparation is to alter the sample to a form that is suitable for final chemical analysis. The number of steps in the sample preparation method depends on the concentration of the analyte in the sample, complexity of the sample, and the limit of quantitation (LOQ) and linear range of the analytical separation method (e.g., GC). Some of the earliest sample preparation methods is simple dilution and extraction (e.g., liquid-liquid extraction (LLE)). In the case of extraction, this method allows the analyte of interest to be extracted from a sample matrix with optimum yield and selectivity [33]. A multiple of techniques have been used for the sample preparation of alkenylbenzenes in variety of foods and related products prior to GC analysis. These include solvent dilution/extraction, LLE, LPME, SPE, QuEChERS, and distillation (steam and hydro-).

#### 2.2.1. Solvent Dilution/Extraction

Addition of an appropriate solvent to the sample is the simplest way for sample preparation prior to GC analysis. For example, hexane was used as dilution solvent in the determination of methyleugenol in tea tree oils. To 5 µL of oil in 2 mL GC vials, 1 mL of n-hexane and 50 mg/L internal standard (IS) (*n*-tetradecane) were added and mixed thoroughly prior to measurement [25]. Meanwhile in another study, hexane was used as extraction solvent in the determination of methyleugenol, eugenol and isoeugenol in fish fillets [30]. However, due to the complexity of the extracts, further sample preparation (clean-up) by solid phase extraction (SPE) was implemented.

The use of ultrasound is known to be useful in diminishing the cost of processing and handling time in the food and chemistry industry. Ultrasound is also a key player in achieving the objective of green chemistry especially in extractions [34]. In ultrasound assisted solvent extraction, the ultrasonic energy passes through a liquid solvent containing the sample in the form of waves. When the waves hit the sample’s surface, it generates a perpendicular or parallel force to the surface. Sonic energy is converted to mechanical energy and presented in the form of shock waves equivalent to several thousand atmospheric pressure. The rapid localised surge in pressure and temperature contributes improved migration of the solvent into the sample. This results in the improved extraction of the analytes [35].

In a recent study for the determination of eight alkenylbenzenes (eugenol, methyleugenol, acetyl eugenol, trans-isoeugenol, safrole, estragole, myristicin and trans-anethole) in pepper and its varieties using GC, a solvent extraction aided by ultrasound was developed. Ethyl acetate (AcOEt) was chosen as extraction solvent because it was suitable for the extraction of non-polar alkenylbenzenes. The procedure was very straightforward, a weighed sample (1 g), AcOEt (20 mL) and dicyclohexylmethanol (10 µL, internal standard) was sonicated at 37 kHz for 45 min at room temperature in an ultrasonic bath. After centrifugation at 3700 rpm (5 min), an aliquot of the AcOEt supernatant was subjected to GC analysis. The mean recovery reported was acceptable in the range of 70% to 120% [20].

#### 2.2.2. LLE

LLE is widely used to transfer an analyte from an aqueous matrix into an extraction solvent. The aqueous matrix and extraction solvent should be immiscible or partially miscible with each other so that the two solvents can be separated easily. Moreover, the analytes from the aqueous matrix should be soluble in the extraction solvent and ideally exhibit high partition coefficients in the solvent. The common extraction solvents used are dichloromethane, chloroform, *n*-butyl chloride, hexane and toluene [36]. An example of an LLE procedure was in the quantification study of methyleugenol in 120 food samples including basil, laurel, tarragon, allspice, nutmeg, lemongrass, cinnamon and anise [32]. In this work, 60% ethanol (that showed highest extraction efficiency) was used as aqueous matrix and methyl tert-butyl ether (MTBE) was used as extraction solvent. Three extraction solvents used for evaluation were 1,1,2-trichloro-1,2,2-trifluoroethane, *n*-hexane and MTBE. MTBE and 1,1,2-trichloro-1,2,2-trifluoroethane showed a similar extraction behaviour (about 90% at first extraction), whereas *n*-hexane had a lower yield (about 65% at first extraction). 1,1,2-trichloro-1,2,2-trifluoroethane was an ozone-depleting chemical; therefore, MTBE was used for further experiments in the study. Cyclodecaneone was used as internal standard (IS) in the experiment to improve the accuracy of quantification. For solid samples, ethanolic homogenate was made by mixing with 100 mL of 60% ethanol. Beverage solution or 1 mL of the ethanolic homogenate was spiked with 200 µL of 40 mg/L cyclodecanone (IS), 5 mL of water and 1 mL of extraction solvent. After shaking and centrifugation, the organic phase was subjected to GC analysis. The recovery for this sample preparation was notable in the range from 95% to 105%.

#### 2.2.3. LPME

LPME which is a miniaturized version of LLE was introduced by Jeannot can Cantwell in the early 1990s [37]. It is designed to be a simple, low cost, high recovery, low solvent consumption and rapid extraction method. LPME is a three-phase extraction method where analytes are extracted from a liquid matrix using a small amount (e.g., tens of microliters) of immiscible extraction solvent with the aid of another solvent. There are three main types of LPME including dispersive liquid-liquid microextraction (DLLME), single-drop microextraction and hollow-fiber LPME [38].

**Figure 3:**
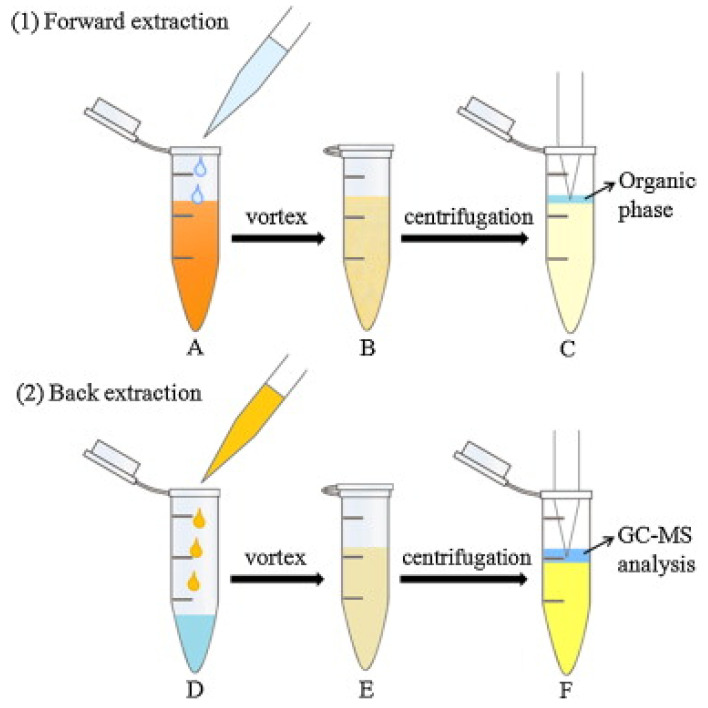
Dual DLLME method (forward and back DLLME) for alkenylbenzenes in oil. Forward DLLME (**1**): (**A**) The *n*-hexane and extraction solution (containing TX-100) were injected into the oil sample; (**B**) a cloudy solution was obtained; (**C**) the supernatant was transferred to another tube. Back DLLME (**2**) (**D**) water and AcOEt were added to the resulting supernatant; (**E**) a turbid solution was obtained; (**F**) the organic phase was mixed with internal standard, for GC-MS analysis. Reprinted with permission from the Authors [26].

An innovative dual DLLME [39] was used for the enrichment of six phenylpropenes, including anethole, estragole, methyleugenol, eugenol, safrole and myristicin in oil samples [26]. In DLLME, a dispersant is used to help the extractant to form fine droplets in the sample solution. The dispersant and extractant are typically organic solvents. The formation of fine droplets increases the extraction efficiency by increasing the contact between extractant and analytes [39]. The procedure for the dual DLLME (forward and back DLLME) method for the alkenylbenzenes is shown in Figure 3. In the first (forward) DLLME, a biodegradable surfactant (TX-100) was used to reduce the surface tension of oil by adsorption at the liquid–liquid interface, increasing the dispersion of extraction phase (*n*-hexane) into the sample oil phase. The organic phase was then subjected to the second (backward) DLLME in order to remove the surfactant which was detrimental during GC-MS analysis. In the backward DLLME, water and ethyl acetate (dispersant) were added to the hexane extract, and the surfactants were removed into the water phase. The efficiency of the dual DLLME method was determined by calculating the enrichment factor which were 4.5, 9.1, 9.8, 16.7 and 37.1× for estragole, eugenol, methyleugenol, safrole and myristicin, respectively [26].

#### 2.2.4. QuEChERS

QuEChERS was initially established to detect pesticide residues in fruits and vegetables but had now rapidly gained attention in the extraction of analytes from different matrices. It is a very popular technique with thousands of papers published according to PubMed [40]. QuEChERS involves two simple steps. The first step is sample preparation and extraction. During this step, the sample is homogenised and an internal standard is added to enhance quantification accuracy. Various acids, salts and buffer are also added to improve the extraction efficiency. Second step is sample extract clean-up. The solvent extract from the first step is cleaned-up using dispersive solid-phase extraction to eliminate possible interfering compounds especially from food extracts [41].

QuEChERS was recently used in the determination of methyleugenol in food samples [31]. For both solid and semi-solid food samples, the sample (2 g) was weighed and placed in a 15 mL centrifuge tube. Methyleugenol-D_3_ (100 µL) and AcOEt (10 mL) was added, and the mixture was vortexed (1 min). Anhydrous magnesium sulphate (2 g) and sodium chloride (0.5 g) was added and the resulting mixture was sonicated for 10 min. After centrifugation, 1 mL of supernatant was collected for clean-up. This was by adding anhydrous magnesium sulphate (500 mg) and primary/secondary amine PSA (100 mg). PSA is a solid phase extraction adsorbent used for sample clean-up, which is available commercially. After the vortex and centrifugation steps, the sample was filtered through a 0.22 µm polytetrafluoroethylene (PTFE) syringe filter and analysed by GC. Satisfactory recoveries were obtained in the range of 94.3–100.3% by this method [31]. In another study aimed to determine flavouring substances in food, a similar extraction method was developed but was less time consuming, straightforward and matrix-independent. The samples (2.5 g) were weighed in a plastic centrifuge tube and spiked with dicyclohexylmethanol (25 µL, IS). AcOEt (5 mL) was added, and the mixture was shaken at room temperature for 30 min. Subsequently, magnesium sulfate (2 g) and sodium chloride (0.5 g) were added. The mixture was then shaken by hand and centrifuged (3500 rpm) for 10 min. 0.5 mL of supernatant were collected and filtered with a mini-uniprep PTFE filter. The recoveries for this extraction method were considered acceptable within the range of 70–120% [28], which was a little poorer than the first extraction method described.

#### 2.2.5. SPE

SPE uses a liquid phase and a solid phase to separate analytes from a solution. It is often used to clean-up the sample before final chemical analysis. In general, the procedure starts by loading a sample solution into a SPE column, and undesired components are removed by flushing with a non-eluting solvent. An appropriate (eluting) solvent is loaded into the column to wash off the desired analytes that were retained in SPE material during the initial steps.

A phenyl-containing group SPE material was used to clean-up the hexane extract of fish fillets that potentially contained methyleugenol, eugenol and isoeugenol [30] (see Section 2.2.1). The SPE column was preconditioned with hexane and AcOEt. It was then flushed with hexane (clean-up) and then AcOEt was used to elute the targeted analytes for GC analysis. The authors examined the efficiency of hexane, AcOEt, acetonitrile (ACN) and dichloromethane (DCM) to elute targets from phenyl SPE columns. They found that hexane was not able to elute eugenol, isoeugenol and methyleugenol but was able to wash off the interferences from the fish fillet samples during SPE. In contrast, AcOEt, ACN and DCM were able to elute the analytes from the phenyl SPE columns. However, AcOEt was chosen to elute the analytes because ACN and DCM can produce adverse effects on human health [42]. Overall, the proposed solvent extraction and SPE clean-up methodology produced acceptable recovery values for methyleugenol, eugenol, isoeugenol from 76.4% to 99.9%. The methodology is also free from rotary evaporation and nitrogen blowing.

#### 2.2.6. Distillation

According to the type of interaction between the water and/or steam, there are two main variants for extraction using distillation: steam distillation and hydro-distillation. Steam distillation has been used for a long time in the analysis of volatiles from high-water-content foods, beverages and essential oils [43,44]. It employs steam and/or water as extracting agent to vaporise the volatile analytes from the raw material. The volatile analytes are vaporised by absorbing heat from the steam. The vapor phase resulted from the process is cooled and condensed. Based on their immiscibility, water separates from the organic phase. As a result, two products are formed which are called the volatile oil (upper phase) and the hydrosol (bottom phase). In dry steam distillation, the steam is forced to flow through a supported matrix. This variant enables the steam to be heated above the boiling point and thus becomes superheated steam. In direct steam distillation, the matrix is inserted above the water in the boiling flask, supported by perforated grid or screen [45]. Steam distillation had been conducted by Muráriková, et al. [27] on basil essential oil which contains alkenylbenzenes.

**Figure 4:**
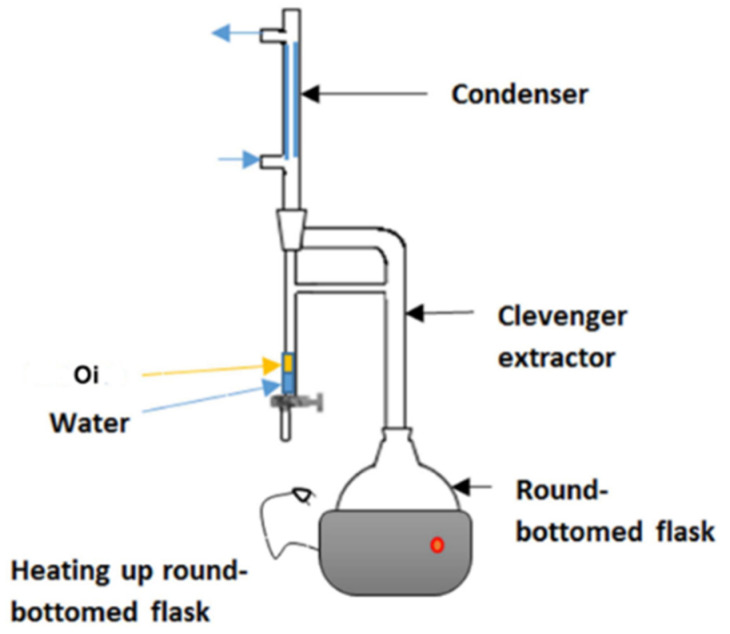
Clevenger apparatus used for hydrodistillation. Reprinted with permission from the Authors [47].

Hydro-distillation is a common extraction method used in GC analysis. In hydro-distillation, there are three main physicochemical processes involved: hydro-diffusion, hydrolysis and decomposition by heat [46]. Clevenger apparatus is the equipment employed in hydro-distillation as shown in Figure 4. The name was titled after its inventor, Joseph Franklin Clevenger in 1928. The round-bottomed flask at the bottom contains the mixture of material and water. As the steam rises, the steam assembles in the condenser and the condensate falls into a burette. In the burette, oil floats (Oi) on the water. After few hours of extraction, the oil can be collected for further use [47].

Since 2010, eight articles described hydro-distillation for the extraction of alkenylbenzenes in various types of food related samples. In the analysis of methyleugenol present in *Cymbopogon khasianus* Hack., Gogoi, et al. [24] collected fresh plant materials that were shade dried for 24 h. Shade drying results in significant increase in essential oil contents [48], most likely by the significant reduction of the water content. In the extraction of alkenylbenzenes in essential oil, the amount of sample used ranged from 50 g to 300 g and the distillation time were carried out between 2 to 3.5 h. The extracted oils were collected and treated with anhydrous sodium sulfate to remove the water in the oil. Subsequently, the samples were stored at 4 °C until GC analysis [17,18,21,22,23,24,29]. Joy, Berle, Affolter and Pegg [18] showed the average recovery was 85.3 ± 3.2% using hydrodistillation technique in their study. An interesting study by Niculau, et al. [49] used the Clevenger type apparatus for the hydro-distillation (at 110 °C for 2 h) of *Pimenta pseudocaryophyllus* essential oil which contained chavibetol and methyleugenol. Semi-preparative HPLC was then used to purify chavibetol and methyleugenol from the crude essential oil. GC with FID was used to determine the purity, with chavibetol (98.7%) having a higher purity compared to methyleugenol (85.3%). The purification method of hydrodistillation method coupled with semi-preparative HPLC can provide chavibetol with high purity and recovery. HPLC will be discussed in the next section, but for analysis.

## 3. HPLC

### 3.1. Analysis

The development of modern HPLC has been commonly linked to the research achievements of Prof. C. Horvath of Yale University. HPLC is one of the most important analytical separation techniques nowadays. A HPLC system consists of five main components: pump, injector, column, detector, data acquisition and control system. The pump provides constant and continuous flow of liquid mobile phase throughout the system. The sample is injected at the injector port and enters the column. The column contains a stationary phase, and it is where the separation of the components in the mixture happens. Once the components exit the column, it enters the detector and produces signals. Data acquisition and control system is a computer-based system that monitors all HPLC parameters [50].

HPLC was used to determine different types of alkenylbenzenes in various plants (e.g., *Cinnamomum zeylanicum* Blume), foods and commercial herbal formulations as shown in Table 2 [51,52,53]. Table 2 also summarizes the sample preparation used, amount of alkenylbenzenes found in the sample, HPLC conditions, detectors, and pertinent reference. All studies except one used reversed phase HPLC, where the stationary phase is non-polar (e.g., C18 stationary phase column) and the mobile phases are typically water-organic solvent (e.g., ACN) mixtures. On the other hand, normal phase HPLC uses a polar stationary phase and the analytes are eluted with a non-polar mobile phase (e.g., with hexane). A hexane and methanol containing mobile phase was used in the normal phase HPLC of *Pimenta pseudocaryophyllus* [49]. Normal phase separations are normally avoided due to the chemical waste generated by the mobile phase used. Interestingly, there were three studies conducted on ultra-performance liquid chromatography (UPLC) using stationary phase particle size of 1.7 µm [13,54,55]. UPLC is an ultra-modern version of conventional HPLC. The main different between HPLC and UPLC is particle size of the solid stationary phase. UPLC uses particle size less than 2 µm therefore it is faster, and results to better resolution and high sensitivity [56]. The common mobile phase used in the determination of alkenylbenzenes by HPLC/UPLC contained different percentages of ACN and water with 0.1% trifluoroacetic acid. ACN is popularly used because it has low viscosity, high elution strength and high chemical stability [57]. However, for the advancement of green analytical chemistry, future studies should replace ACN with the bioderived and safe organic solvent methanol [57]. Methanol on the one hand has a higher viscosity and lower eluting strength compared to ACN.

**Figure 5:**
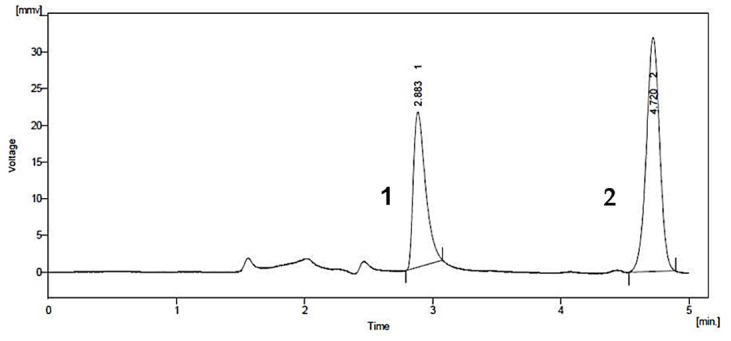
Chromatogram of boswellic acid (**1**) and myristicin (**2**) by HPLC with UV-VIS detection. Reprinted with permission from the Authors [52].

**Figure 6:**
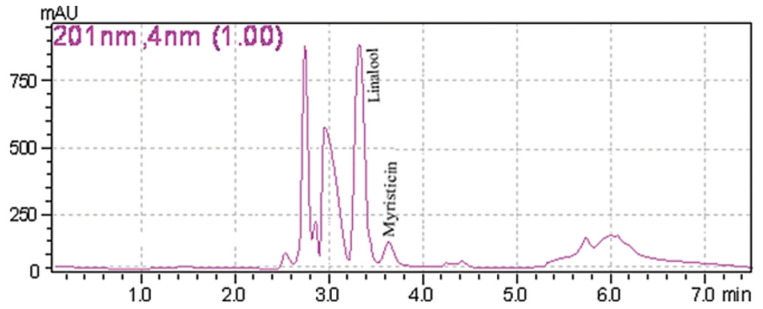
Chromatogram of myristicin and linalool in a plant by HPLC with DAD. Reprinted with permission from the Authors [53].

A common detector employed in HPLC is based on photometry, i.e., ultraviolet-visible (UV-VIS)/diode array detector (DAD). UV-VIS/DAD is highly applicable to alkenylbenzenes because these compounds contain a UV active chromophore (see Figure 1, e.g., benzene ring in their structures). The studies considered (see Table 2) did not implement mass spectrometry detection most likely because alkenylbenzenes are neutral compounds which are not ionisable with common ionisation sources used in mass spectrometry (e.g., electrospray ionisation). In UV-VIS/DAD detection, visible and UV light passes through a flow cell. The mobile phase that contains the analytes passes through the flow cell, and a plot of signal intensity and time can be constructed (chromatogram). Quantitation from the obtained signal intensities can be done using one or more wavelengths. DAD can measure the absorption at different wavelengths and the components can be identified from the spectra obtained. HPLC with photometry was reported in the determination of boswellic acid and myristicin present in commercial herbal formulation [52]. A sample chromatogram obtained from the standards is shown in Figure 5, where the two targeted analytes were well separated within a short period of time (~5 min). Another fast HPLC separation (~7 min) of two alkenylbenzenes in plants was done by Lung, Stan, Opriş and Soran [53] as shown in Figure 6. The myristicin and linalool peaks were well resolved from each other, as well as the other components in the real sample.

### 3.2. Sample Preparation

#### Solvent Extraction

Many studies evaluated the use of different solvents such as hexane, methanol and ethanol for the solvent extraction of secondary metabolites in various plant parts (e.g., leaves and seeds) [59]. They have found that highly polar solvents like methanol gave better recoveries. This was the most likely reason leading to the authors’ decision to use methanol as extraction solvent during the determination of alkenylbenzenes using HPLC. In fact, during this review period, only solvent extraction was used in conjunction with HPLC. First, Gursale, et al. [51] used methanol in the solvent extraction of alkenylbenzenes in *Cinnamomum zeylanicum* Blume. Recovery of methyleugenol was 99% after solvent extraction with sonication followed by simple filtration. The recovery value was considered acceptable because the recovery value was within 80% to 110% (see FDA Guidelines for the Validation of Chemical Methods) [60]. This study was an inspiration for later studies. For example, Alajlouni, et al. [58] used methanol to extract alkenylbenzenes from plant food supplements. The recoveries found for estragole, myristicin, apiol was 85.3% ± 2.9%, 101.1% ± 5.4% and 94.5 ± 7.6%, respectively. Suparmi, et al. [13] determined alkenylbenzenes in Indonesian jamu. The recoveries found for methyleugenol, elemicin, safrole, myristicin and estragole were 103.8% ± 0.1%, 108.1% ± 1.8%, 105.6% ± 23.0%, 96.1% ± 1.3%, 99.7% ± 3.4, respectively. However, solvent extraction with methanol cannot extract all alkenylbenzenes with good repeatability (e.g., safrole with ~20% error).

In the determination of myristicin in pesto sauce, Al-Malahmeh and co-workers [55] used the methanol extraction method of Ávila and co-workers [61] with few modifications. Originally, Ávila and team macerated dry samples (e.g., basil leaves) with only 15 mL methanol for 12 h at 50 °C. The sample was filtered and wash with water and methanol. The filtrates were collected and quantitatively diluted and then stored at 5 °C [61]. In the Al-Malahmed and team extraction method, a larger volume of methanol (i.e., 80 mL) was used for maceration. The resulting samples were centrifuged for 5 min and an aliquot of the supernatant was stored at −20 °C [55]. They used the margin of exposure (MOE) approach to evaluate the potential risk of myristicin from intake of pesto sauce. Based on the levels of myristicin found in the pesto sauces studied, >30 g consumption of pesto sauce on a daily basis will achieve MOE levels that will warrant priority for risk management.

Meanwhile, the study by Lung et al. [53] was interested to know the effect of microwave irradiation on the levels of linalool and myristicin in plants. The plants were exposed to microwave irradiation prior to extraction. Similar to methanol extraction, the sample was soaked in the solvent, sonicated and then filtered prior to HPLC analysis. No explanation or systematic studies on other solvents were conducted, however very high recovery for myristicin was achieved between 98%–98.48%. Interestingly, they found that the concentration of myristicin in plants was significantly affected by microwave irradiation, but not linalool.

## 4. Capillary Electrophoresis

Capillary electrophoresis (CE) is an electric field driven analytical technique used for the separation of compounds in a mixture [62,63]. In CE, a fused silica capillary is filled with a separation media (e.g., buffer), and the nature of the separation media defines the mode of the separation. In the CE mode where a buffer is used a separation media, only charged analytes are separated. The separation mechanism is based on differences in the electrophoretic mobility of the analytes. The general procedure in CE is as follows, after conditioning the capillary with separation media, sample is injected at one end of the capillary, a high voltage is applied at both ends of the capillary that is dipped into vials that contain the separation media. The analytes migrate to the detector at the other side of the capillary via electrophoretic mobility and electroosmotic flow. Detection is typically using on-line (on-capillary) with a UV/VIS detectore. Another mode of CE is micellar electrokinetic chromatography (MEKC), where the addition of a pseudostationary phase (e.g., micelles) to the buffer allows the separation of both neutral and charged analytes. The pseudostrationary phase acts similarly to the stationary phase in HPLC. The components in the mixture separate based on the partition differences of the analyte between aqueous buffer solution (mobile phase) and micelles (pseudostationary phase) [64,65]. Neutral analytes gain an ‘effective’ electrophoretic mobility due to its interaction with a charged pseudostationary phase. MEKC is the suitable CE mode for alkenylbenzenes separation because these analytes are electrically neutral (see Figure 1).

**Figure 7:**
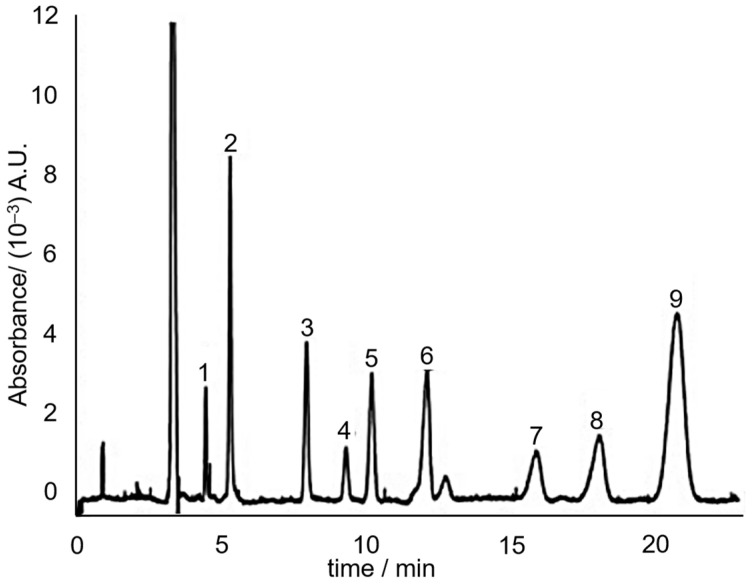
MEKC electrochromatogram of sassafras essential oil. Peaks: (3) eugenol; (6) methyleugenol; (7) safrole; (8) myristicin. Reprinted with permission from the Authors [66].

Indeed in 2008, Huhn, et al. [66] utilized the principle of MEKC to separate the hydrophobic neutral alkenylbenzenes eugenol, safrole, methyleugenol and myristicin. The separation media consisted of 60 mM sodium dodecyl sulphate (SDS) in 7.5 mM sodium borate (pH 9). SDS forms micelles above its critical micelle concentration (which is ~3 mM in the presence of salts [67]). Three separation media additives that improved the separation were urea, ACN and calcium chloride. The study concluded that addition of urea and ACN makes it possible to reduce the retention factor of hydrophobic solutes without compromising separation efficiency. The addition of CaCl_2_ allowed to fine-tune the electroosmotic flow without influencing the effective electrophoretic mobility. A sample electrochromatogram of the separation of the sassafras essential oil components, including the targeted alkenylbenzenes is shown in Figure 7.

## 5. Conclusions

This review highlighted the different analytical separation methods (GC, LC, CE) for alkenylbenzenes analysis from mainly between 2010 and 2020, with GC and LC as the main players. The most popular sample preparation method for GC was hydrodistillation whereas solvent extraction with methanol was typical in LC. Hydrodistillation was applicable for extraction of essential oils but not for food samples. Due to the complexity of food matrices, extraction techniques such as LLE, SPE and QuEChERS have been developed. Methanol extraction is simple with high recoveries but may suffer from matrix interferences during the analytical separation. We expect new developments for extraction that reduces matrix interferences while increasing detection sensitivity. During the review period, various alkenylbenzenes were found in many food and food related products. Analysis of alkenylbenzenes will remain to be an important research topic for guarding our public health, since these compounds are (or potentially) carcinogenic and genotoxic. The availability of alkenylbenzenes in food is huge, due to the many sources of these compounds from various plants used mainly as spice or flavouring.

Most of the methods were able to perform analysis of the targeted samples. For example in GC, the analysis were satisfactory using one dimension only, while two dimensional GC separation are applied for more complicated samples [68]. For LC, the separations were done using standard columns that uses up to 1 mL/min of mobile phase. This could generate significant volumes of chemical waste due to organic solvents used in the mobile phase. However, there are smaller columns that are currently being developed that minimises mobile phase usage (e.g., open-tubular columns) [69]. Translation of methods from standard columns to smaller scale columns can be foreseen in the future. In addition, CE does not generate significant volume of chemical waste but only 1 paper was published in this area. Therefore, modern analytical methods that are faster, greener (less chemical waste generated from analysis), and more accurate should be developed for determining the occurrence of alkenylbenzenes in various food and related products.

## Figures and Tables

**Table 2:** Application of HPLC/UPLC in the study of alkenylbenzenes.

Analytes	Sample/s	Sample Preparation	Amount of Alkenylbenzenes Found in Sample/s	HPLC/UPLC Conditions	Detectors	Ref.
methyleugenol	*pimenta pseudocaryo-phyllus*	hydrodistillation	methyleugenol (27.9 mg/g)	mobile phase:hexane (A), ethanol (B) column:Phenomenex Luna amino (4.6 mm × 150 mm × 10 µm) condition:isocratic condition with ratio hexane and ethanol at 92:8 flow rate: 1 mL/min injection volume:10 µL wavelength: 230 nm LOD: not reported LOQ: not reported	UV-VIS	[49]
methyleugenol	*cinnamomum zeylanicum blume*	methanol extraction	methyleugenol (0.5 mg/g)	mobile phase:water (A), ACN & methanol (B) at ratio 45:20:35 column: reversed-phase C_18_ Intersil ODS-3V-C_18_ (150 mm × 4.6 mm × 5 µm) condition:isocratic with the ratio of methanol:ACN:water is 35:20:45 flow rate:1 mL/min injection volume: not reported wavelength: 221 nm LOD: 0.10 µg/mL (methyleugenol) LOQ: 0.30 µg/mL (methyleugenol)	UV-VIS	[51]
myristicin	commercial herbal formulation	methanol extraction	myristicin (0.3 mg/g) ^†^	mobile phase: water (A), ACN (B) column:Supelco 516 C_18_ (250 mm × 4.6 mm × 5 µm) condition: ACN:water is 85:15 flow rate: 1 mL/min injection volume: 20 µL wavelength: 205 nm LOD: 0.63 µg/mL (myristicin) LOQ: 1.91 µg/mL (myristicin)	UV-VIS	[52]
myristicin	plants	*n*-hexane-diethyl ether extraction	myristicin (66.3 µg/mL)	mobile phase:water (A), ACN (B) column: LiChrosorb RP-18 (250 mm × 4 mm × 5 µm) condition:start from 0 to 1 min with 100%B, decrease to 25%B in 15 min. flow rate: 1 mL/min injection volume: 5 µL wavelength: 201 nm LOD:32.68 µg/mL (myristicin) LOQ: 64.57 µg/mL (myristicin)	DAD	[53]
estragole myristicin apiol	plant food supplements	methanol extraction	estragole (17.2 µg/g) myristicin (26.0 µg/g–1804.5 µg/g) apiol (93.0 µg/g–6486.6 µg/g)	mobile phase: water with 0.1%TFA (A), ACN (B) column: Waters Acquity C_18_ (50 mm × 2.1 mm × 1.7 µm) condition: start at 31% ACN, keep at 31%ACN for 5 min, increase to 80%ACN over 4 min and keep for 1 min, decrease to 0% over 1.5 min and keep for 1 min, increase back to 31%ACN. flow rate: 0.6 mL/min injection volume:not reported wavelength:209 nm (apiol, myristicin), 201 nm (estragole) LOD: not reported LOQ: not reported	DAD	[58]
elemicin methyleugenol myristicin safrole apiol estragole	Indonesian jamu	methanol extraction	elemicin (not found) methyleugenol (4.8 ± 1.6 µg/g–128.6 ± 0.9 µg/g) myristicin (33.9 ± 7.2 µg/g–440.1 ± 24.8 µg/g) safrole (3.8 ± 0.5 µg/g–18.8 ± 3.2 µg/g) apiol (not found) estragole (13.3 ± 1.3 µg/g–23.9 ± 6.3 µg/g)	mobile phase: water with 0.1%TFA (A), ACN (B) column: Waters Acquity UPLC BEH RP 18 (25 mm × 2.1 mm × 1.7 µm) condition: start at 30.5%ACN for 15 min, increase to 80%ACN over 1 min, keep at 80%ACN for 0.5 min, decrease to 0%ACN over 1.5 min, keep at 0%ACN for 1 min and back to 30.5%ACN. flow rate:0.6 mL/min injection volume: 3.5 µL wavelength: 206 nm (elemicin), 202 nm (methyleugenol, safrole), 210 nm (myristicin, apiol) and 225 nm (estragole) LOD: actual *w/w* values were not reported LOQ: actual *w/w* values were not reported	DAD	[13]
methyleugenol myristicin estragole apiol	pesto sauce	methanol extraction	methyleugenol (22.9 ± 3.1 µg/g–56.4 ± 7.5 µg/g) estragole (3.2 ± 1.5 µg/g–34.1 ± 2.8 µg/g) myristicin (13.2 ± 1.2 µg/g–15.8 ± 0.0 µg/g) apiol (3.4 ± 0.2 µg/g)	mobile phase: water with 0.1%TFA (A), ACN (B) column: Acquity UPLC BEH C_18_ (50 mm × 2.1 mm × 1.7 µm) condition:isocratic at 40%ACN for 4 min flow rate:0.6 mL/min injection volume: 3.5 µL wavelength: 201 nm (methyleugenol, estragole), 210 nm (myristicin, apiol) LOD: not reported LOQ: not reported	DAD	[55]

^†^ Converted from % (*w*/*w*) to mg/g using the formula: mg/g = (x/100) × 1000, where x = value in percentage.

## Data Availability

No new data were created or analyzed in this study. Data sharing is not applicable to this article.
